# Germline and somatic mutations in histologically atypical congenital hyperinsulinism

**DOI:** 10.3389/fendo.2025.1692539

**Published:** 2026-01-05

**Authors:** Annette Rønholt Larsen, Evgenia Globa, Ditte Caroline Andersen, Catarina Limbert, Åsa Löfgren Mattsson, Anne Lerberg Nielsen, Michael Bau Mortensen, Eva Kildall Hejbøl, Klaus Brusgaard, Sönke Detlefsen, Henrik Thybo Christesen

**Affiliations:** 1 Hans Christian Andersen Children’s Hospital, Odense University Hospital, Odense, Denmark; 2 Department of Clinical Research, Faculty of Health Sciences, University of Southern Denmark, Odense, Denmark; 3 Odense Pancreas Center (OPAC), Odense University Hospital, Odense, Denmark; 4 Department of Clinical Genetics, Odense University Hospital, Odense, Denmark; 5 Department of Pediatric Endocrinology, Ukrainian Scientific and Practical Center of Endocrine Surgery, Transplantation of Endocrine Organs and Tissues of the Ministry of Health of Ukraine, Kyiv, Ukraine; 6 Andersen-group, University of Southern Denmark, Odense University Hospital, Dep. Clinical Biochemistry, Odense, Denmark; 7 Unit for Pediatric Endocrinology and Diabetes, Hospital Dona Estefania, Lisbon, Portugal; 8 Department of Pediatrics, Helsingborg Hospital, Helsingborg, Sweden; 9 Department of Nuclear Medicine, Odense University Hospital, Odense, Denmark; 10 Upper Gastrointestinal and Hepato-Pancreato-Biliary Section, Department of Surgery, Odense University Hospital, Odense, Denmark; 11 Department of Pathology, Odense University Hospital, Odense, Denmark; 12 Steno Diabetes Center, Odense University Hospital, Odense, Denmark; 13 Department of Clinical Genetics, Lillebaelt Hospital, Vejle, Denmark

**Keywords:** congenital hyperinsulinism, genetics, histology, *HK1*, hyperinsulinemic hypoglycemia, laser-capture microdissection, mosaicism

## Abstract

**Background:**

In histologically atypical congenital hyperinsulinism (CHI), correlations between clinical, histological and genetic features are largely unknown. Laser-capture microdissection may be used to identify low-grade mosaic DNA variants in the islets of Langerhans.

**Aim:**

To investigate genotype-histotype-phenotype correlations histologically in atypical CHI.

**Methods:**

In our single-center cohort of hyperinsulinemic hypoglycemia (HH) patients, 77 underwent pancreatic surgery. In those with histologically atypical CHI, genetic analyses included sequencing of frequent CHI genes from blood and bulk pancreatic tissue and tests for Beckwith-Wiedemann Syndrome (BWS) where appropriate. If negative, a targeted 140-gene panel including the non-coding region of *HK1* was performed in blood, pancreatic bulk tissue and islets isolated by laser-capture microdissection. Histological, immunohistochemical and morphometric analyses were performed on pancreatic tissue.

**Results:**

The 77 HH patients were classified histologically as K_ATP_-channel focal CHI (n=48), K_ATP_-channel diffuse CHI (n=14), insulinoma (n=6), non-insulinoma HH in teenagers (n=1), BWS (n=1), unclassified (n=2). Histologically atypical CHI patients (n=5/70; 7.1%) had a median (range) birth weight of 2965 (2650-3385) grams and a clinical disease onset at 93 (1-259) days. 18F-DOPA PET/CT showed diffuse tracer uptake. In three patients, genetic analysis showed *HK1* intron 2 variants, of which one was present in germline (*de novo* heterozygous) while the other two had somatic low-grade mosaic alterations in bulk pancreatic tissue (n=1) or exclusively in islets after isolation by laser-capture microdissection (n=1). Patient 4 showed a *CACNA1D* frameshift mutation suggesting Cav1.3-channel gain-of-function properties. No relevant genetic changes were found in Patient 5. In all five atypical CHI specimens, pancreatic histology showed slight changes with areas having pronounced occurrence of large islets of Langerhans, while small islets and endocrine cell clusters were evenly distributed. Giant cell nuclei were observed, but at much lower frequencies compared to K_ATP_-channel diffuse CHI.

**Conclusion:**

Histologically atypical CHI was seen in 7.1% of surgically treated CHI patients and characterized by discrete changes with enlarged islets of Langerhans and a low frequency of giant nuclei in endocrine cells. Genetics showed heterozygous or low-grade mosaic *HK1* intron 2 DNA variants in three patients. Low-grade mosaic pancreatic genetic changes may only be detectable after islet isolation by laser-capture microdissection.

## Introduction

Congenital hyperinsulinism (CHI) is a rare disease affecting 1:28,000 to 1:40,000 infants (1), characterized by abnormally high insulin secretion from the endocrine pancreas, leading to hyperinsulinemic hypoglycemia (HH). CHI is a heterogeneous disorder with varying clinical presentation, histology, genetics, and treatment response. Non-syndromal CHI has been associated with mutations in a least nine different key genes, namely *ABCC8*, *KCNJ11*, *GCK*, *GLUD1*, *HADH*, *HK1*, *HNF1A*, *HNF4A*, and *SLC16A1* (2), of which inactivating mutations in the KATP-channel genes *ABCC8* and *KCNJ11* are the most common, accounting for about 90% of the diazoxide-unresponsive CHI patients (3, 4). In diazoxide-responsive patients, the genetic basis is known in only 22–35% of affected patients (4, 5). CHI usually presents during the newborn period and is the most common cause of severe and persistent hypoglycemia in infancy (6). At least 28 syndromes are associated with CHI, most frequently Beckwith-Wiedemann syndrome (BWS) (7).

CHI has two major histopathological forms: diffuse CHI or focal CHI, with each distinct genotype–histotype correlation. Classic histological diffuse CHI is caused by biallelic mutations in *ABCC8* or *KCNJ11.* K_ATP_-channel diffuse CHI is seen in 39–58% of the patients undergoing surgery and affects the entire pancreas (3, 8). K_ATP_-channel focal CHI is associated with a paternally inherited *ABCC8 or KCNJ11* mutation plus a somatic loss in the maternal 11p15 region, resulting in paternal uniparental disomy (pUPD) (9). This two-hit condition causes loss of heterozygosity in the K_ATP_-channel and an imbalance of the imprinted growth-related genes *H19*, *IGF2*, and *CDKN1C*, leading to focal adenomatosis with confluent hyperplastic islets in the focal lesion. Focal CHI is seen in 42–51% of patients with surgery for CHI (3, 8). Rare variants of K_ATP_-channel focal CHI include multiple focal or very large lesions as recently reviewed (10).

Other genetic forms of CHI are more challenging to categorize histologically, as few such patients have undergone surgery. Patients with activating *GCK* mutations in germline have a histological pancreatic picture ranging from normal to increases in islet size, or slightly increased size of single beta cell nuclei (11–14). In a few surgically treated patients with *GLUD1* mutations, a few beta cell nuclei had a moderate increase in size (15–17). In BWS-CHI, mosaic changes in chromosome 11p15 leads to segmental or diffuse excessive growth of pancreatic islets (18).

Morphological mosaicism of the pancreatic islets, showing larger islets of Langerhans restricted to a few lobuli and smaller islets distributed throughout the pancreas, has been described by Sempoux et al. in a few atypical patients (19), with subsequent identification of mosaic genetic expression of *HK1*, or somatic *GCK* mutations, in some of them (20).

Localized islet nuclear enlargement (LINE) has been described in other few patients (3) with later identification of low-grade somatic mosaic mutations in *ABCC8* or *GCK* (21). Scattered nuclear enlargement in a few endocrine cells in a few islets localized to a certain area in the pancreas has been described in few patients with genetic mosaicism in *GLUD1* or *GCK* (22, 23), of which two with low-grade somatic *GCK* mutation had occasional enlarged and confluent islets (23). Mutation in a non-coding region of *HK1* and rare occurrence of nuclear enlargement in endocrine cells has been demonstrated in one patient (24).

The clinical term “atypical CHI” may represent any atypical manifestation, e.g. atypical clinical onset, or atypical (inconclusive) tracer uptake by 18F-DOPA PET/CT. From the histological viewpoint, atypical CHI histology can be negatively defined as a pancreas specimen from a patient with HH not consistent with diffuse CHI, focal CHI, insulinoma, or excessive growth of endocrine cells like that observed in CHI associated with BWS.

We aimed to investigate the genotype-histotype-phenotype correlations in an HH cohort of surgically treated nationally and internationally referred patients with atypical CHI histology, i.e. non-K_ATP_-channel diffuse or focal CHI and non-BWS histology, including genetic analysis of islets of Langerhans isolated by laser-capture microdissection.

## Patients, materials and methods

### Setting and patients

At the International Hyperinsulinism and Hypoglycemia Center, Hans Christian Andersen Children’s Hospital, Odense University Hospital, Denmark, a cohort of 190 patients with documented HH were evaluated, 77 of which underwent pancreatic surgery and histological evaluation. The diagnosis of HH was based on an **i**nsulin level >1.25 μU/mL (8.7 pmol/L) or C-peptide >0.5 ng/mL (>0.17 nmol/L) during hypoglycemia (6).

**Figure 1:**
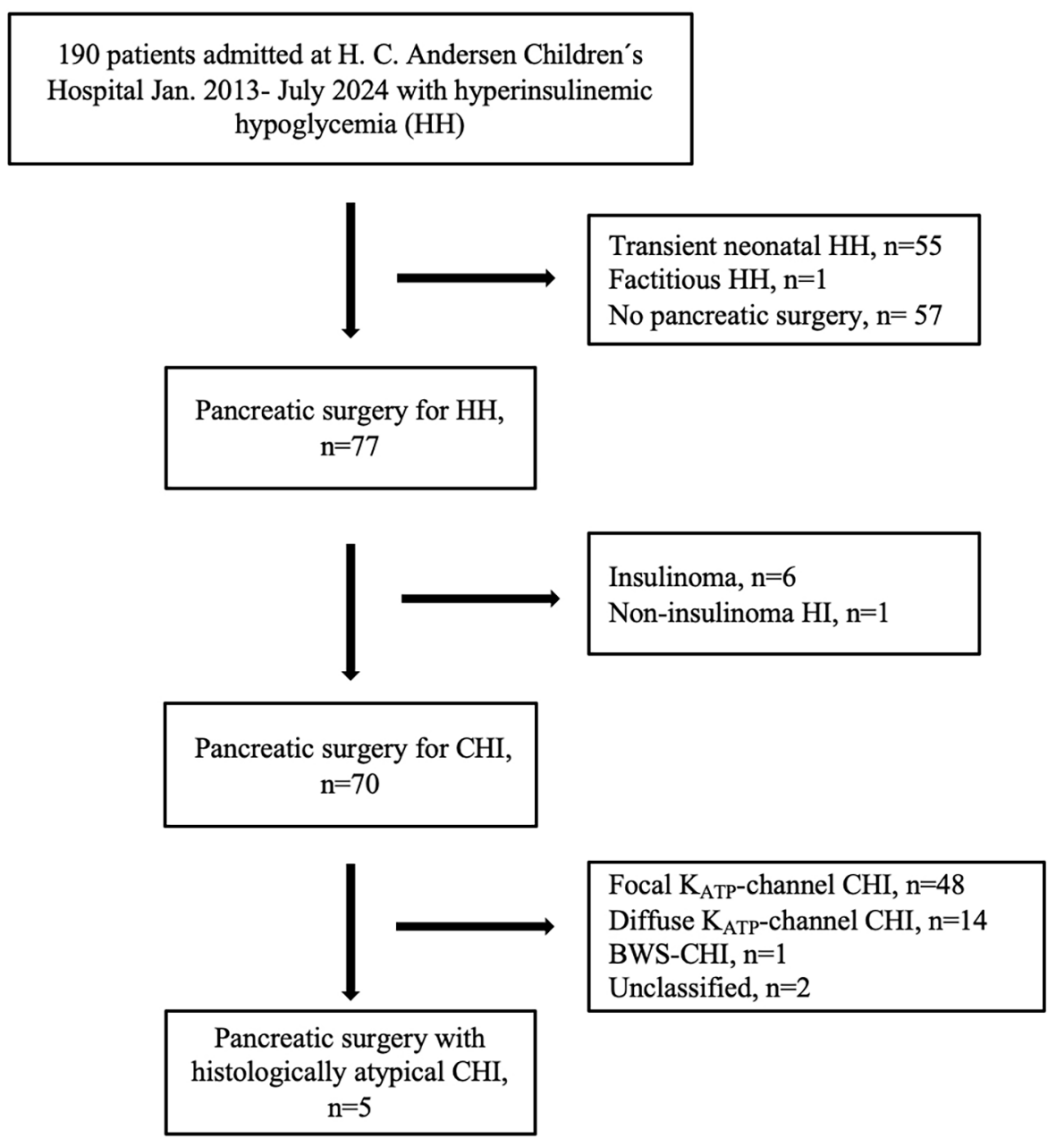
Patient inclusion flow chart for histologically atypical congenital hyperinsulinism (CHI).

The 77 pancreatic histology HH cases were classified as insulinoma (n=6), non-insulinoma HI in teenagers (n=1), and 70 with the following CHI subtypes: KATP-channel focal CHI (n=48), KATP-channel diffuse (n=14), atypical CHI (n=5), BWS-CHI (n=1) and unclassified (no final histology) (n=2), **Figure 1**.

All surgical specimens were examined by the same pathologist with expertise in pancreatic pathology (SD). In this study, histologically atypical CHI was defined as CHI without the classical histological features of K_ATP_-channel diffuse CHI, K_ATP_-channel focal CHI or BWS-related CHI. HH was diagnosed based on unsuppressed serum insulin during non- or hypo-ketotic hypoglycemia by use of a sensitive insulin assay (6, 25). Routine genetics were performed including the at the time available sequencing panel as previously described (26). 18F-DOPA PET with diagnostic CT scan was performed prior to surgery as previously described (27). In patients with atypical histology, clinical characteristics were retrospectively retrieved from their hospital files at Odense University Hospital with follow-up by contacts to their referring physicians (EG, CL, ÅML) in their home countries. Clinical details of Patient 2, 4, and 5 have previously been described in a Ukrainian CHI cohort report (28).

### Immunohistochemistry

Tissue specimens were analyzed by microscopy of hematoxylin-eosin (H&E) staining of formalin-fixed, paraffin-embedded 4 µm thick sections; immunohistochemical staining using the BenchMark Ultra immunostainer (Ventana Medical Systems, Tucson, AZ) with the OptiView-DAB detection kit (Ventana Medical Systems, Tucson, AZ); nuclear counterstaining with the BenchMark Ultra instrument using Hematoxylin II (Ventana Medical Systems, Tucson, AZ) and coverslipping using a Tissue-Tek Film coverslipper (Sakura, Alphen aan den Rijn, The Netherlands). For this project, all tissue specimens underwent additional immunohistochemical examination, which included H&E staining, synaptophysin, chromogranin A, insulin, glucagon, somatostatin and tumor suppressor p57. An overview of the antibodies, dilutions, incubation times and epitope retrieval procedures is provided in **Supplementary Table 1**.

Based on the stainings mentioned above, pancreatic histology and immunohistochemistry of the atypical CHI cases (n=5) was re-evaluated. Special emphasis was on whether there were signs of BWS-related CHI, K_ATP_-channel diffuse or focal CHI, overgrowth of endocrine cells, morphological mosaicism of pancreatic islets, or LINE.

### Digital image analysis

Morphometric analysis was performed on insulin-stained pancreas sections. The sections were scanned on a NanoZoomer slide scanner (Hamamatsu Photonics) and the digital images were analyzed in QuPath v0.4.3 (29). Each specimen was divided in 4–7 areas bases on natural borders. The areas ranged from 6.8 to 26.4 mm^2^. The volume fraction of insulin-positive pixels was determined in each area. For each patient, the area with the highest insulin volume fraction and the area with the lowest insulin volume fraction was selected and the islet sizes were registered and compared. On H&E stained sections, analysis of the size of nuclei was performed in 60–80 islets of Langerhans from each patient, comprising areas with large islets and areas with no or only few large islets. As control, normal surgical pancreatic tissue from a 6-year-old male was used.

### Laser-capture microdissection of islets of Langerhans

The formalin-fixed paraffin-embedded tissue (FFPE) sections were cut at 10 µm on a microtome (Leica, Inc.). The sections were mounted on a 1.4 µm thick PET nuclease-free membrane (MMI, Inc.), de-paraffinized with xylene and rehydrated in 99%, 93%, and 77% alcohol. The tissue was stained on an autostainer LINK 48 (Agilent, Inc.) with insulin antibody, 2D11-H5 (Santa Cruz, sc-8033, Inc), diluted 1: 2000. Incubation lasted 1 hr. Insulin antibody was detected on EnVision FLEX/HRP + Mouse LINK (Agilent K800221, Inc.). An inverted microscope (Olympus, Inc.) was combined with a laser (MMI, Inc.) and linked to a computer. The optimized laser was set up to cut with a velocity of 15 µm/s; laser focus was 355 µm; and laser power was 70.4%. Nuclease-free silicon IsolationCaps (MMI, Inc.) with adhesive lids were used to isolate the tissue. A sterile blade and forceps were used to peel tissue from the cap to Eppendorf tubes. In all patients, islet isolation was performed from representative areas of the resected tissue, blinded to the histo-morphometric analysis.

### Sequencing

A sequencing panel of known CHI genes was performed in DNA from blood and tissue genetically unexplained patients after routine investigation, including *ABCC8*, *KCNJ11*, *GCK, GLUD1, HADH, HNF1A, HNF4A, INS, INSR, SLC16A1* and *UCP2*. Additional tests for BWS were performed in leukocyte DNA and resected pancreatic tissue, as previously described (30).

In those with unexplained genetics in the above-mentioned analyses, the gene CHI gene panel was performed also on representative areas of the resected pancreatic tissue (blinded for the histo-morphometric analysis) in search for somatic mutations. If negative, a targeted 140-gene next generation sequencing (NGS) panel + the non-coding region of *HK1* was performed in leukocyte DNA, pancreatic tissue and isolated islets, the latter to detect possible low-grade mosaicism confined to the endocrine pancreas.

In details, we used the Gene Reads ™ DNA FFPE protocol (Qiagen, Inc.) to purify DNA from both whole tissue and isolated islets of Langerhans from the pancreas of the patients. FFPE DNA was processed using Twist Custom Targeted Panels (Twist Bioscience, Inc.). Unique Molecular Identifiers (UMI) were included in the subsequent adapter, and sequencing of the targeted 140-gene NGS panel + non-coding *HK1* (for gene list, see **Supplementary Table 2**) was performed on NovaSeq 6000 (Illumina, Inc.) with a mean read depth of 1.733 for each target region. The use of UMI allows analysis for mosaicism to a very low variant allele frequency (VAF). By power calculation, we were able to detect a true VAF of 0.5% in 1700 reads with a power of 0.97, or 0.99, given a threshold read of 1 SD, or 3 SD, respectively.

The Dragon somatic pipeline (Illumina, Inc.) was used to process the sequencing data. VarSeq 2.4 (Golden Helix, Inc.) was used for the downstream filtering of the variants. We specifically searched for missense, nonsense, frameshift, and splice variants with a Phred score >10 and a VAF >0.5%. The Genome Aggregation Database v4.0,0 (GnomAD) was used to evaluate the frequency of the variants. For missense variants, the prediction software SIFT (sorting intolerant from tolerant) and Polyphen2.0 (polymorphism phenotyping) values were used. Nomenclature was given according to GRCh38/hg38.

ENCODE cCRE, ENCODE Regulation, CpG, FANTOM5, GeneHancer, GTEx cis-eQTLs, JASPER Transcription Factors, ORegAnno, RefSeq FuncElements, VISTA Enhancers was applied in the UCSC browser to identify regulatory elements in the non-coding region of *HK1.*The ENCODEPROJECT (www.encodeproject.org) was used to analyze for H3K4me3 targets in the endocrine pancreas. RNAseq data from the GEO repository (GSE249790, GSE248349) was used to analyze beta-cell mRNA expression patterns. ESEFinder, release 3.0 (https://esefinder.ahc.umn.edu) was used to analyze for splicing enhancers. The GTEx (https://www.gtexportal.org) database was used to analyze expression patterns of alternative isoforms. Analysis for alternative promotors was performed using data from EPDnew (https://epd.expasy.org/epd/human/human_database.php?db=human) applying filters including data from CAGE, RAMPAGE, FANTOM5, HK1 (ENST00000359426.7).

Lastly, copy number variant (CNV) analysis was performed using Golden Helix Varseq v 2.2.5. The software calls CNV events based on Z-score, ratio-plot, and VAF metrics. Eventual CNVs were computed using Coverage Statistics using the coding regions of hg38 (plus/minus 20 nucleotides in the non-coding regions) with more than 10 samples with less than 20% difference from each individual sample as reference.

## Results

Of the 70 surgically-treated patients with CHI, five surgical specimens (7.1%, 95%C.I. 2.3-15.9%) had histologically atypical CHI, according to the definition used in the present study. In addition, two patients had unclassified histology. One of these only had a small biopsy from the pancreatic head in an area suspicious for focal CHI by 18F-DOPA PET/CT, however not identifiable by the surgeon, intraoperative ultrasound, or by freeze or final microscopy, which was inconclusive. Her germline CHI genetic panel was normal.

The other patient had a known pathogenic paternal *KCNJ11* variant, p.Pro254Leu, and a focal 18F-DOPA uptake in the uncinate process (SUV_max_ ratio 1.7). During surgery, a focal lesion could, however, not be identified, leading to “blind” resection of the uncinate process. The freeze and final histology was inconclusive. No tissue genetics was performed. At post-discharge follow-up, the patient was largely, but not completely, cured.

The five histologically atypical patients had unknown genotype by our routine genetic CHI panel. Genetic investigations for BWS also showed normal results. In the following, their clinical characteristics, histological features and genotype are described in detail.

### Clinical phenotype

**Figure 2:**
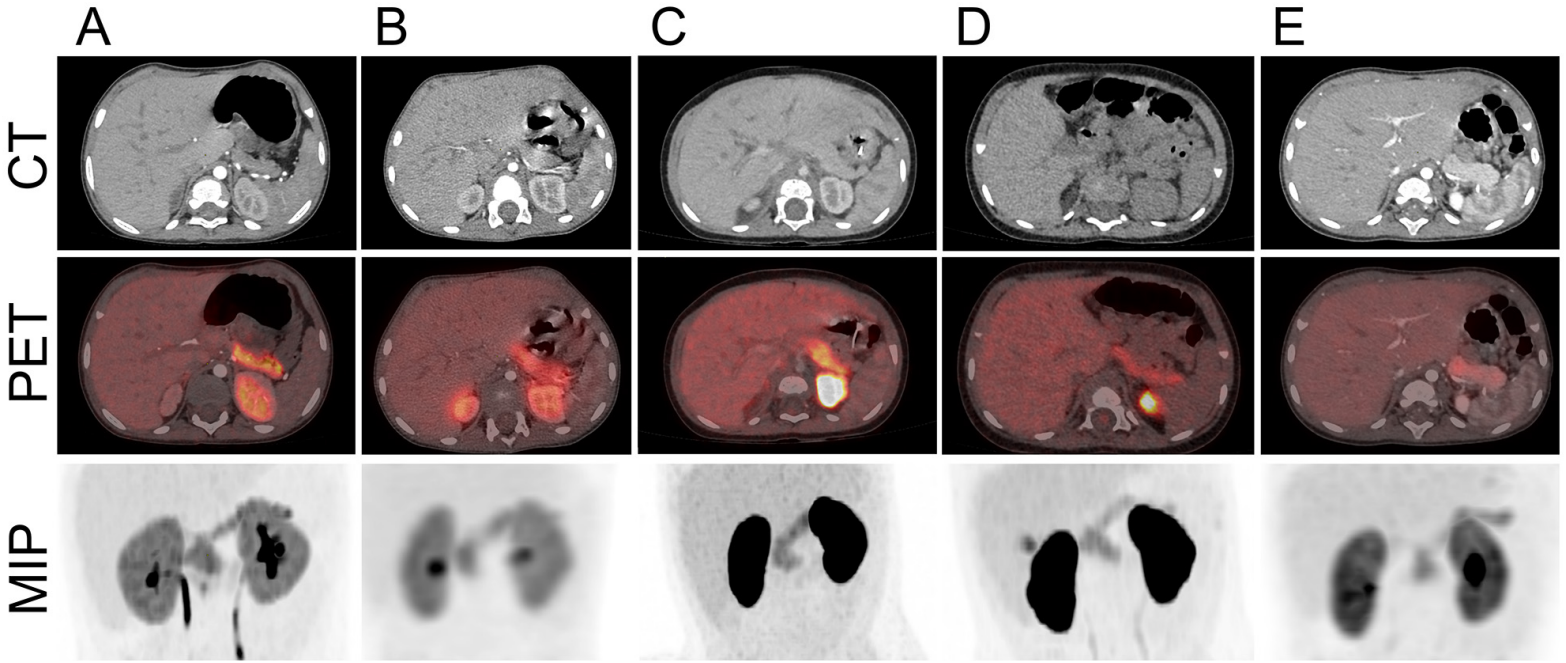
18F-DOPA PET/CT in patients with histologically atypical congenital hyperinsulinism (CHI). **(A)** Patient 1. Diffuse 18F-DOPA tracer uptake, marginally enlarged pancreatic tail. **(B)** Patient 2. Diffuse 18F-DOPA tracer uptake. **(C)** Patient 3. Diffuse tracer uptake, enlarged tail area. **(D)** Patient 4. Diffuse tracer uptake. **(E)** Patient 5. Diffuse tracer uptake, pronounced tail enlargement. CT, computerized tomography; PET, position emission tomography; MIP, maximal intensity projection.

The clinical patient characteristics are presented in **Table 1**. The patients were born at term with a median (range) birth weight of 2965 (2650-3385) g. Their parents were healthy for glucose-related diseases. The clinical onset of hypoglycemia occurred at a median (range) age of 93 (1-259) days. All patients were diazoxide unresponsive. Prior to surgery, on i.v. glucose only, serum insulin values during hypoglycemia were within (n=4) or above (n=1) the normal range of 18–174 pmol/L confirming ongoing HH. By 18F-DOPA PET/CT, all had diffuse tracer uptake and three of the five patients had left-sided enlargement especially in the pancreatic tail (**Figure 2**). The patients underwent surgery at a median age of 17 (8-27) months with resection of 33–85% of the pancreas. At follow-up to a median age of 8.3 (5.6-11.25) years, Patient 1 was treated with diazoxide in gradually decreasing doses from 16.0 to 5.6 mg/kg/day, Patient 2 and 5 had no treatment, Patient 3 had dietary treatment of mild episodes of hypoglycemia, and Patient 4 was at first treated with diazoxide, long-acting somatostatin analogue and glucocorticoids in lower doses that prior to surgery, but only with long-acting somatostatin analogue at last follow-up.

**Table 1:** Summarized clinical characteristics of five patients diagnosed with histologically atypical congenital hyperinsulinism.

Characteristics	Patient 1	Patient 2	Patient 3	Patient 4	Patient 5
Gender	Female	Female	Female	Male	Female
Gestational age (weeks+)	39+	At term	37+	At term	38+
Birth weight (g)	3385	2850	2965	2650	3192
Age at first recorded hypoglycemia (days or months)	Day 1	11 months (symptoms from 4 months)	8.5 months	2.5 months	1 month
Fasting test* Plasma glucose (mM) Insulin (18–174 pM) C-peptide (400-1600 pM) Proinsulin (2.1–13 pM)	2.3 171 1126 39	2.7 343 1737 76	2.7 97 931 39	2.0 52 841 53	2.7 115 874 37
18F-DOPA PET/CT scan of the pancreas	Diffuse labeling, enlarged pancreatic tail	Diffuse labeling	Diffuse labeling, enlarged pancreatic tail	Diffuse labeling	Diffuse labeling, enlarged pancreatic tail
Age at pancreatic surgery	2 years, 3 months	2 years, 3 months	10 months	8 months	13 months
Pancreatic surgery, type	33% pancreatectomy	80% pancreatectomy	50% pancreatectomy	85% pancreatectomy	66% pancreatectomy
Age at last follow-up (years, months)	9 years, 0 months	11 years, 3 months	5 years, 6 months	6 years, 10 months	8 years, 3 months
Treatment at last follow-up	Diazoxide 5.6 mg/kg/day	No treatment. LASA discontinued age 10 years	Dietary. Frequent feeds. Expanded release corn starch and rescue glucose by gastric tube	LASA. Diazoxide and glucocorticoids discontinued at 6 years’ age	None. Normal fasting test at discharge age 14 months. No clinical relapse

*Reference intervals in brackets. LASA, long-acting somatostatin analogue.

### Histological and immunohistochemical features

**Figure 3:**
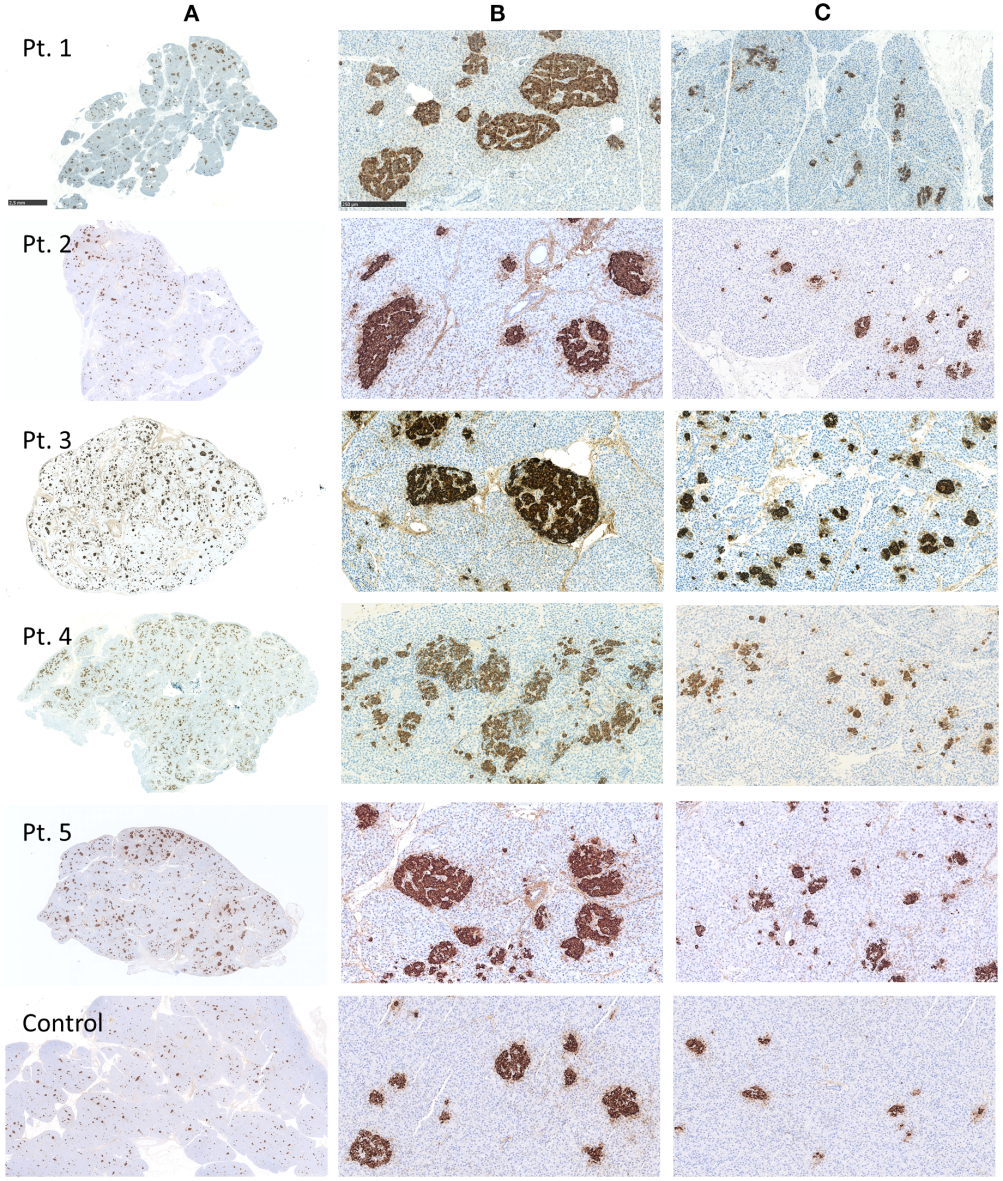
Histology of pancreatic tissue from Patient 1–5 with histologically atypical congenital hyperinsulinism (CHI) and control, immunohistochemically stained for insulin. **(A)** Overviews of the specimens. Scale bar: 2.5 mm. **(B)** Large islets of Langerhans were restricted to certain areas in the resected specimens. **(C)** Smaller islets and clusters of endocrine cells were observed throughout the resected pancreatic tissues. Scale bar for **(B, C)**: 250 µm.

Histologically and immunohistochemically, the pancreas specimens from the five patients showed only discrete changes, different from K_ATP_-channel diffuse CHI, K_ATP_-channel focal CHI and BWS-related CHI. In all five pancreatic specimens, larger islets of Langerhans were found in certain areas in the pancreas, while smaller islets and clusters of endocrine cells were observed throughout the resected pancreatic tissue (**Figure 3**).

### Morphometry using digital image analysis

**Figure 4:**
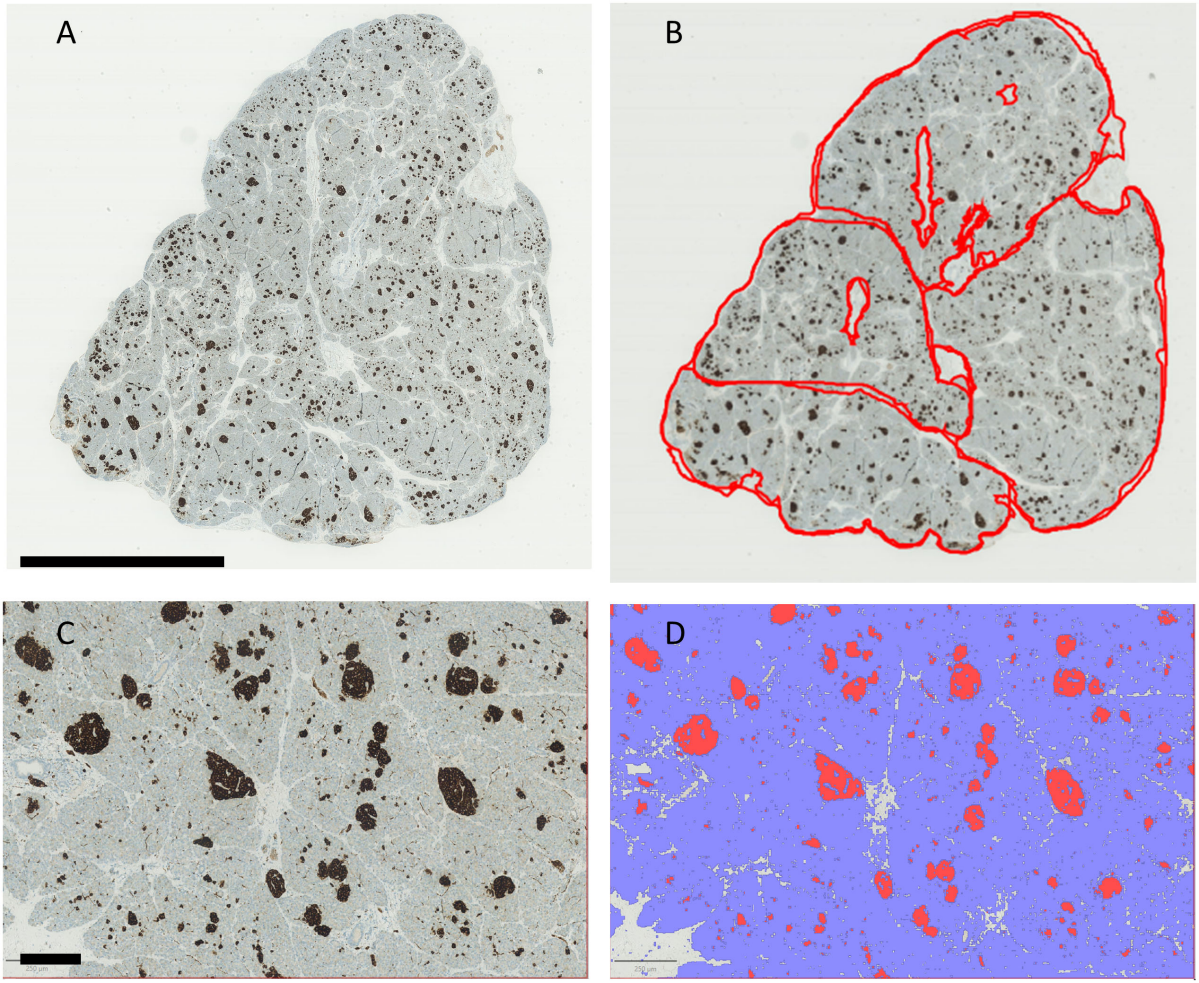
Quantification of pancreatic tissue stained for insulin in Patient 5. **(A)** shows the unlabeled section. **(B)** shows the specimen divided into four separate areas. The major blood vessels have been excluded from the analysis. **(C)** shows beta cells stained positively for insulin (dark brown). In **(D)**, the same area is analyzed with QuPath for detection of insulin positive cells (red) and exocrine pancreatic tissue (blue). Scale bar for **(A, B)** = 5 mm; scale bar for **(C, D)** = 250 µm.

Morphometric analyses of insulin-stained pancreatic specimens from Patient 1–5 showed a higher beta-cell volume fraction in the patients than in the control (**Table 2**, **Figure 4**). An impression of larger islet variation in the patients was confirmed, when dividing the specimens in smaller parts according to natural septae (**Figure 4**). We hereby detected a higher variation in islet quantity and islet size between the different parts of the specimens in the patients vs. control (**Table 2**, **Figures 3**, **4**).

**Table 2:** Morphometric data of insulin-stained pancreatic specimens from patient 1–5 and a non- congenital hyperinsulinism control.

Pancreatic tissue	Patient	1	2	3	4	5	Patient 1–5 median (range)	Control
Whole specimen	Size (mm^2^)	67.9	100.5	96.5	97.0	72.8	96.5 (67.9-100.5)	218.4
	Beta cell volume fraction (%)							
	Volume fraction	2.9	3.0	8.0	7.1	9.3	7.1	2.1
	Range*	1.8 - 5.6	1.6 - 5.4	7.1 - 9.4	5.3 - 9.5	5.1 - 16.2	2.9-9.3	1.5-2.6
Areas with large islets (highest beta cell volume fraction)	Islets per mm^2^	18.9	13.7	38.7	18.7	41.6	18.9 (13.7-41.6)	10.4
	Islet size, top 10% (mm^2^)							
	mean	0.029	0.018	0.017	0.015	0.018	0.018 (0.015-0.029)	0.012
	minimum	0.012	0.009	0.005	0.005	0.006	0.006 (0.005-0.0120)	0.007
	maximum	0.105	0.049	0.079	0.044	0.049	0.049 (0.044-0.105)	0.021
Areas without large islets (lowest beta cell volume fraction)	Islets per mm^2^	23.3	25.1	49.3	43.4	51.2	43.4 (23.3-51.2)	8.9
	Islet size, top 10% (mm^2^)							
	mean	0.008	0.005	0.005	0.007	0.007	0.007 (0.005-0.008)	0.008
	minimum	0.003	0.003	0.002	0.003	0.003	0.003 (0.002-0.003)	0.005
	maximum	0.017	0.009	0.03	0.024	0.023	0.023 (0.009-0.03)	0.014

*Range of the volume fractions determined in subareas of the section as visualized in **Figure 4**.

When comparing islet morphology in low and high beta cell volume areas, this heterogeneity was further detailed. Thus in all patients, the number of islets/mm^2^ was higher in areas without large islets compared to areas with large islets (median 43.4 vs. 18.9 islets/mm^2^). In the control, this variation was not evident (8.9 vs. 10.4 islets/mm^2^). In all patients, the mean islet size (top 10%) was higher in areas with high beta cell volume (0.018 mm^2^) compared to areas with low beta cell volume (0.007 mm^2^). A similar pattern was seen in the control but with less variation (0.012 mm^2^ vs. 0.008 mm^2^). The atypical CHI patients were thus characterized by having a high beta cell volume, in some areas due to large islets and in other areas due to many small islets and with a generally larger variation in islet size and number, compared to the control.

**Figure 5:**
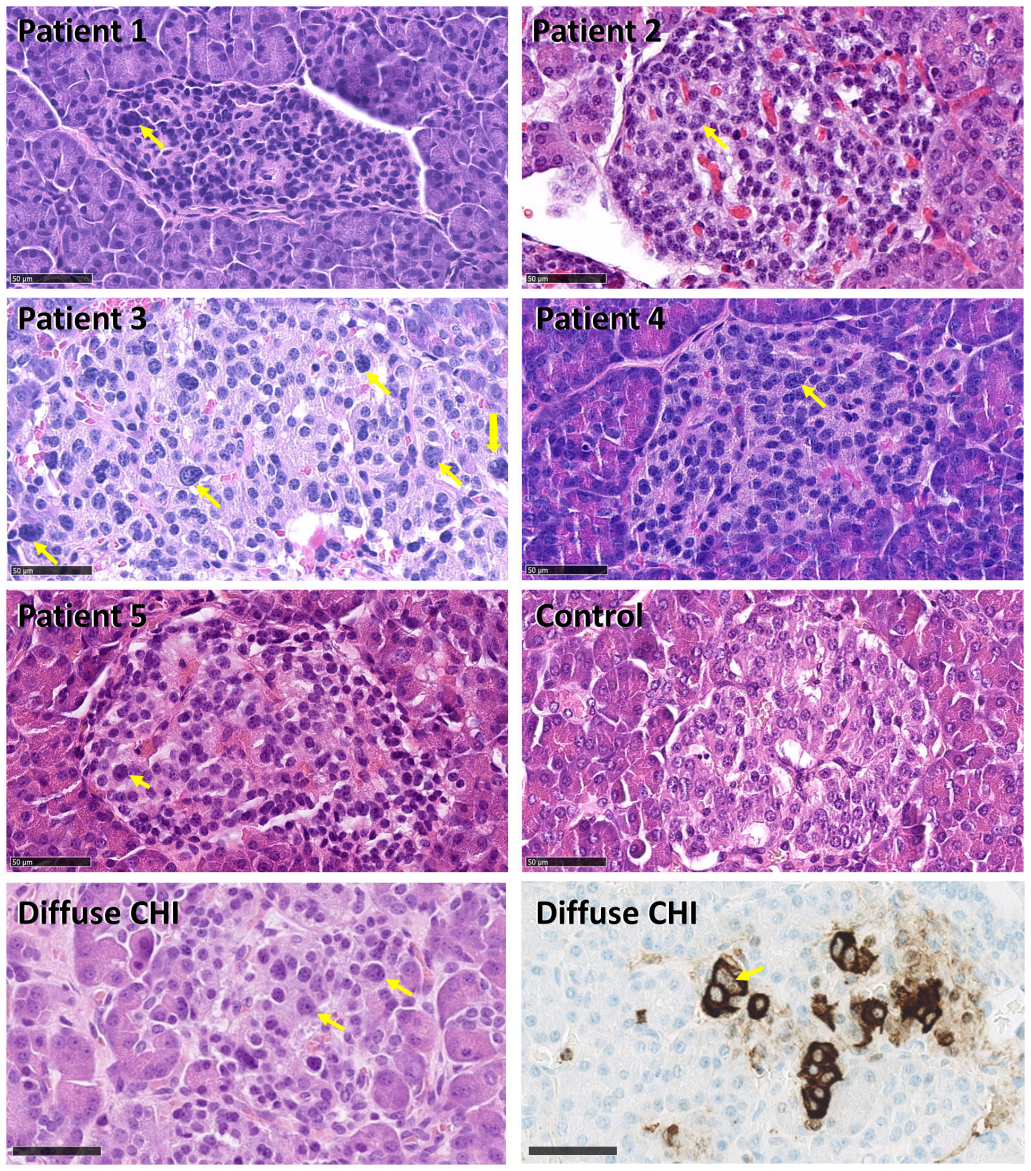
Histology of pancreatic islets in histologically atypical congenital hyperinsulinism (CHI) and diffuse CHI. Examples of nuclei size in islets of Langerhans. The arrows point to nuclei larger than 75 µm^2^, which corresponds to three times the average size of acinar nuclei. In the 5-m-old patient with diffuse CHI, large nuclei are shown in an islet, and in an area with diffuse beta cell distribution (stained for insulin), respectively. Scale bars: 50 µm.

Nuclei larger than three times the size of acinar cell nuclei were seen in islets of Langerhans in all patients but most pronounced in Patient 3 and mostly in larger islets. The number of giant nuclei per area of islet tissue was 2.4 per mm^2^ in Patient 1, 10.4 per mm^2^ in Patient 2, 24.4 per mm^2^ in Patient 3, 13.5 per mm^2^ in Patient 4, and 3.7 per mm^2^ in Patient 5. No giant nuclei were detected in the control (**Figure 5**).

### Genotype

In Patient 1, we identified a *de novo* germline heterozygous non-coding *HK1* variant in position 10:g.69,348,891C>T (**Table 3**, **Supplementary Figure 1**). In Patient 2, the same non-coding *HK1* variant was found in low-grade mosaicism with a VAF of 1.3% in leukocyte DNA, 1.2% in whole pancreatic tissue and 0.9% in isolated islets. In Patient 3, another low-grade *HK1* somatic variant in position 10:g.69,348,896A>T was found exclusively in in the isolated islets of Langerhans with a VAF of 1.9%. No patients or control had a non-coding *HK1* variant below the VAF cut-off 0.5% in any of the analyses.

**Table 3:** Overview of the genetic findings in patient 1–5 with histologically atypical congenital hyperinsulinism.

Specimen	Analysis	Patient 1	Patient 2	Patient 3	Patient 4	Patient 5
Blood	11 gene panel*	Neg	Neg	Neg	Neg	Neg
	140 gene panel**	Neg	Neg	Neg	*CACNA1D* c.4638_4642delAGCGT Paternal heterozygous	Neg
	*HK1* non-coding region, intron 2	10:g.69,348,891C>T Heterozygous *de novo*	10:g.69,348,891C>T Low-grade mosaicism VAF: 1.3%	Neg	Neg	Neg
Whole pancreatic t issue	11 gene panel	Neg	Neg	Neg	Neg	Neg
	CNV	Neg	Neg	Neg	Neg	Neg
	140 gene panel	Neg	Neg	Neg	*CACNA1D* c.4638_4642delAGCGT Paternal heterozygous	Neg
	*HK1* non-coding region, intron 2	10:g.69,348,891C>T Heterozygous	10:g.69,348,891C>T Low-grade mosaicism VAF: 1.2%	Neg	Neg	Neg
Islets of Langerhans, by LCM	140 gene panel	Neg	Neg	Neg	*CACNA1D* c.4638_4642delAGCGT Paternal heterozygous	Neg
	CNV	Neg	Neg	Neg	Neg	Neg
	*HK1* non-coding region, intron 2	10:g.69,348,891C>T Heterozygous	10:g.69,348,891C>T Low-grade mosaicism VAF: 0.9%	10: g.69,348,896A>T Low-grade mosaicism (Negative in whole tissue) VAF: 1.9%	Neg	Neg

*ABCC8, KCNJ11, GCK, GLUD1, HADH, HNF1A, HNF4A, INS, INSR, SLC16A1 and *UCP2*.

**Full gene list shown in **Supplementary Table 2**.

CNV, Copy number variants; LCM, Laser-capture microdissection; VAF, Variant allele frequency.

**Figure 6:**
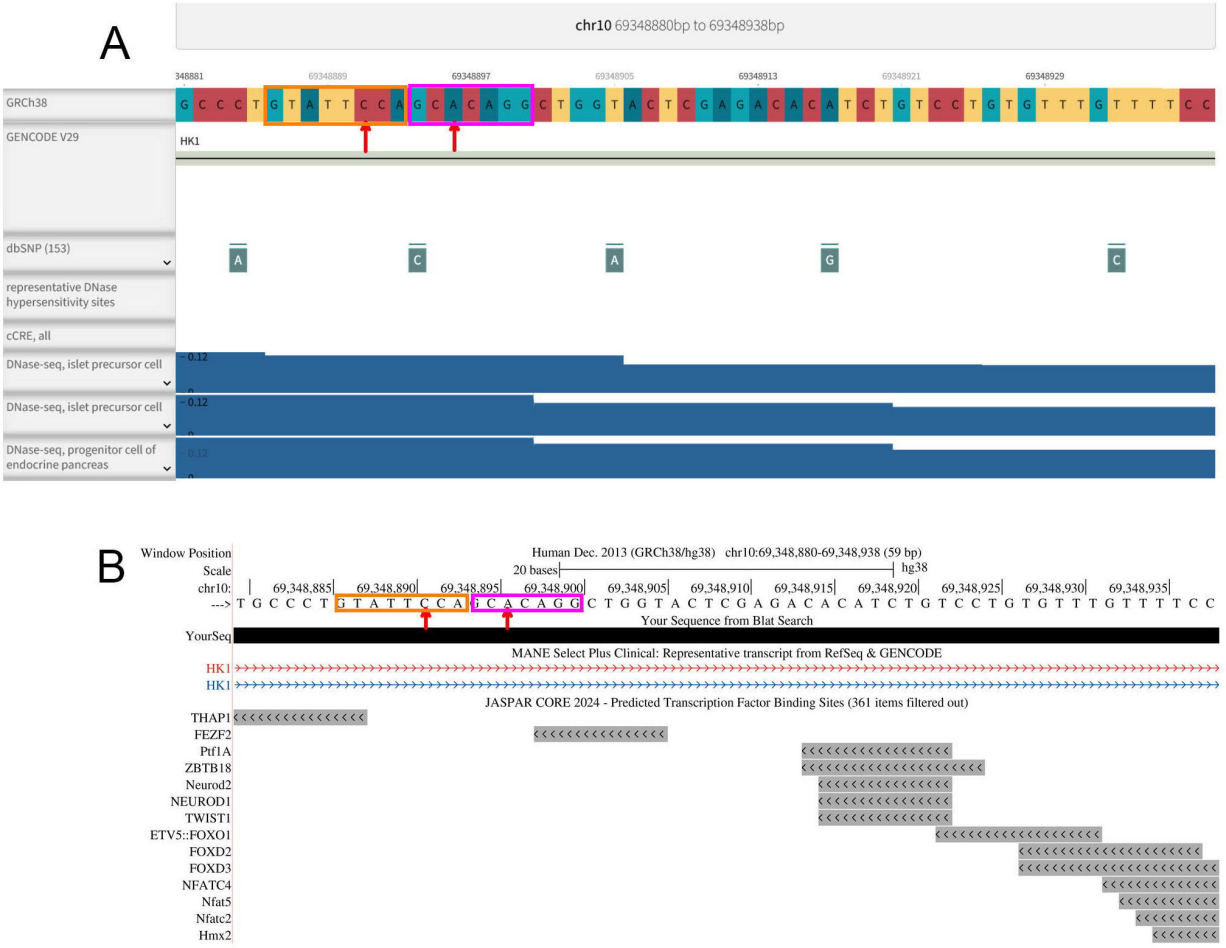
Lack of transcriptional binding factor sites in the non-coding *HK1* region. **(A)** Analysis for DNase hypersensitive sites using ENCODEPROJECT data. **(B)** JASPER analysis for regulatory elements. The two non-coding *HK1* variants are shown with red arrows. The SF2/ASF and SRp40 sites are indicated with orange and magenta boxes, respectively.

**Figure 7:**
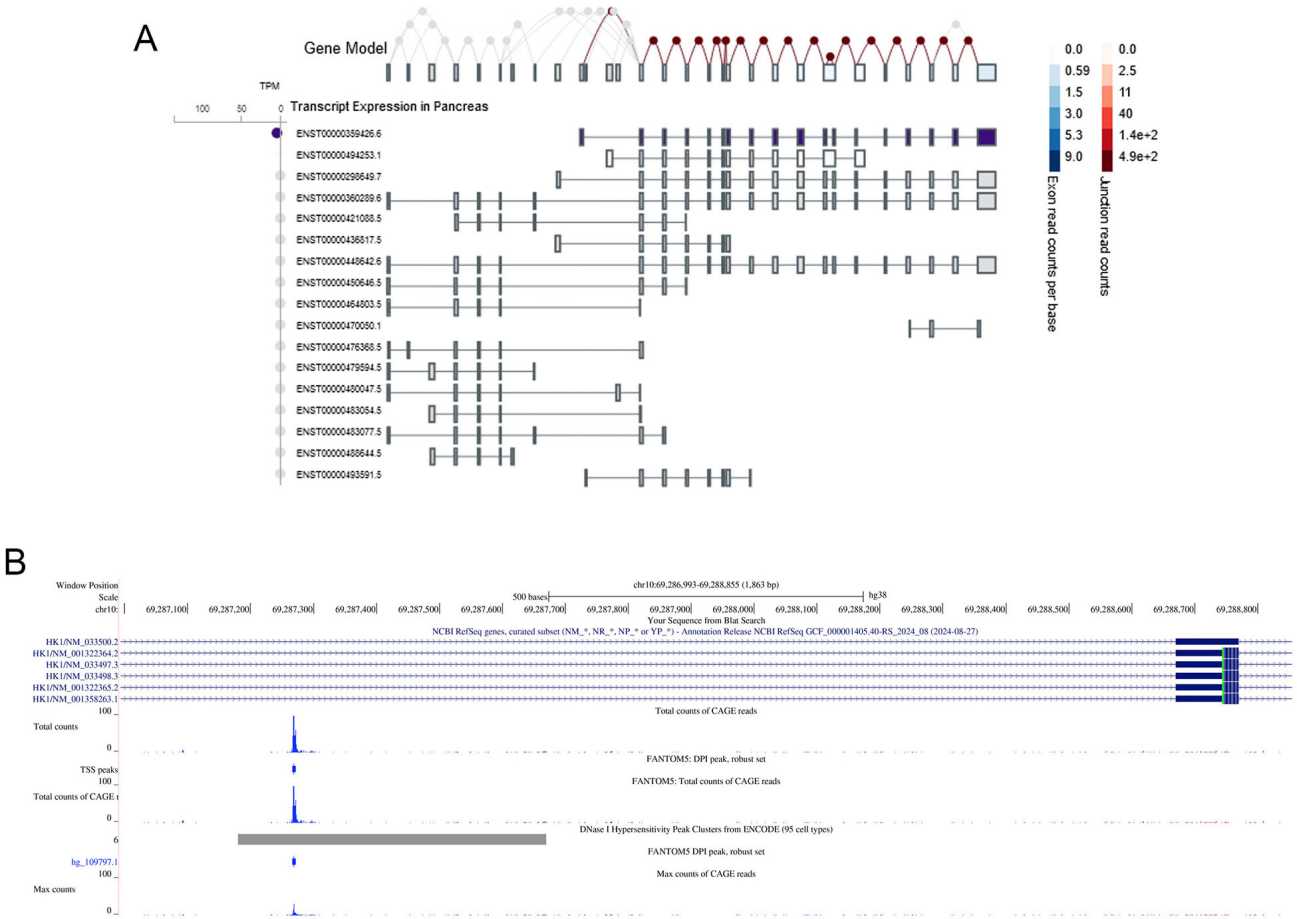
Alternative isoforms and promotor usage of HK1. **(A)** Using the GTEx portal for HK1 the normal splicing pattern of the pancreas expressed HK1 can be seen (ENST00000359426.6). As is illustrated exon 2 is an ubiquitously expressed exon thus changing the splicing pattern of this exon will lead to alternative promoter usage. **(B)** Data from EPDnew applied in the UCSC browser to illustrate an possible alternative promoter usage at HK1 (ENST00000359426.7) exon 2.

Data on the two non-coding *HK1* intron 2 variants did not point to regulation at the transcriptional level (**Figure 6A**). No DNase hypersensitive sites were found in the area with the *HK1* variants reported. Analysis using the filters in UCSC did not point to the presence of regulatory elements in the target region (**Figure 6B**). Rather, ESEfinder showed the *HK1* variants to be situated in potential exon splicing enhancers for SRSF2 (SC35) and SRSF1 (SF2/ASF) (**Figure 7**). Both non-coding *HK1* variants caused the ESE activity to be down-regulated, 10:g.69,348,891C>T splice site score from 2.71 to 0.0, and 10:g.69,348,896A>T splice site score from 3.78 to 2.66, suggesting alterations in the expression of *HK1* isoforms.

In Patient 4, a novel paternal heterozygous frameshift *CACNA1D* variant was found (**Table 3**). The father of Patient 4 had no glucose disorders, but arterial hypertension since youth with normal aldosterone/renin ratio. In Patient 5, no variants of interest were detected.

## Discussion

In a cohort of 70 patients surgically treated for CHI, five (7.1%) had atypical histology without features characteristic of K_ATP_-channel diffuse or focal CHI, or BWS-CHI. Histological analysis showed occurrence of larger islets (mean size 0.02 mm^2^) in certain areas, while smaller islets (mean size 0.007 mm^2^) were distributed throughout the pancreas. This was somewhat reminiscent of the so-called morphological mosaicism of pancreatic islets as per Sempoux et al. However, the larger islets were not entirely restricted to one or a few lobules (19). In addition, enlarged nuclei were found in a few large islets in each case, but at a much lower frequency than in classical K_ATP_-channel diffuse CHI. In three patients, non-coding *HK1* variants were found in heterozygous or low-grade mosaic form, one of the latter only detectable in isolated pancreatic islets. Analysis of the two identified non-coding variants suggested alternative splicing with altered isoform expression of *HK1*.

### Relative frequency

The relative frequency of atypical histology in surgically treated CHI patients has, to our best knowledge, not been reported in the literature. Our relative frequency of 7.1% was higher than expected based on the relative few reports of histologically atypical patients from large CHI cohorts. Moreover, two unclassified patients possibly had histologically atypical CHI, one with a possible low-grade somatic mosaic CHI mutation without germline representation, the other with the possibility of a novel variant of focal CHI: A paternal *KCNJ11* germline mutation *plus* a second somatic hit of low-grade mosaic pUPD11p15 in an area with clear focal 18F-DOPA uptake, but absence of a distinct focal lesion by surgery and histology; more simply named “low-grade mosaic focal CHI”. Others have reported a VAF <50% in somatic genetic investigations of focal lesions (31).

As for other hyperinsulinism expert centers, referral bias may play a major role as also seen by the high number of focal CHI patients in our cohort owing to preference of reference of international patients with paternal K_ATP_-channel mutation predicting focal disease.

### Genotype-phenotype correlation

The five patients had a lower median birth weight and a later clinical presentation compared to K_ATP_-channel diffuse or focal CHI patients from our (26, 32) and other (3, 4) cohorts. Our Patient 1 with a heterozygous *de novo HK1* variant had early-onset CHI from day 1 and a higher birth weight compared to the Patients 2 and 3 with a somatic low-grade mosaic *HK1* variant, a relatively lower birth weight and a later clinical presentation. In the study of Wakelin et al. (24), the 17 individuals had an early clinical onset with an interquartile range from birth to 14 day’s age and a heterozygous or high-grade mosaic (17 and 30%) *HK1* variant in blood. In the recent study of Bennett et al. on 1761 genetically unsolved CHI patients (33), 5% (n=89) had germline variants (including variants of unknown significance) in the proposed cis-regulatory region of *HK1* with large phenotype (birth weight, disease onset and severity) variations for those with likely pathogenic or pathogenic variants. Only six not previously reported patients had mosaic *HK1* variants, counted in 12-34% of the leukocytes. These relatively high-grade mosaic HK1-CHI patients had a median (range) birth weight of 3590 (3490-4400) g and a diagnosis of HI from birth to 13 weeks of age. Their reported heterozygous patients had an median (range) birth weight for term babies of 3536 (1810-5190) g and a diagnosis of hyperinsulinism ranging from day 1 to 26 years; the diagnosis probably not reflecting the clinical onset of first hypoglycemia.

Although large phenotype variations occurs for dominant HK1-CHI as for GCK-CHI, and seemingly indistinguishable from high-grade mosaic HK1, it is reasonable to assume that patients with somatic low-grade mosaic *HK1* variants tend to have a less severe phenotype without macrosomia and overt hypoglycemia at birth. Yet, *HK1* expression, which is normally disallowed in the beta cells, may cause significant fasting hyperinsulinism even in low-grade mosaic HK1-CHI, in keeping with the very high glucose affinity of hexokinase 1 (*K*_m_*<*0.05 mM) (34), compared to a physiological *K*_m_ of 5 mM for glucokinase (hexokinase 4).

By 18F-DOPA PET/CT, mild enlargement of the left (tail) part of the pancreas was clinically observed in Patient 1 and 3. This did not correlate with specific genetic or histological endocrine findings and may represent normal variation.

In our Patient 4, we identified a novel heterozygous *CACNA1D* frameshift mutation. *CACNA1D* encodes the long-acting (L-type) voltage-gated calcium channel Cav1.3 alpha1 subunit, which is expressed in several cell types including the beta cell. Normal-functioning Cav1.3 channels control calcium influx to the beta cell during membrane polarization. Previous studies have identified activating *CACNA1D* mutations in very few patients with CHI (35, 36). In CACNA1C-CHI, both activating and inactivating properties have been found (37). Truncating mutations in the alpha 1 subunit of several *CACNA1* genes with dominant-negative effects have been reported (38). The functional role of our patients’ heterozygous frameshift mutation remains to be established.

Our *CACNA1D* truncating mutation was of paternal origin and the father had no glucose disorders. This may be attributed to variable penetrance or unidentified mutations in other genes.

*CACNA1D* mutations cause arterial hypertension in rats (39) and a *CACNA1D* polymorphism is linked to increased blood pressure in white humans (40), independent of aldosterone, in keeping with the father’s arterial hypertension despite a normal aldosterone/renin ratio. Prolonged follow-up of our Patient 4 is thus warranted to detect eventual hypertension at a later age.

Our Patient 5 had a relatively late onset of hyperinsulinism and the most pronounced enlargement of the pancreatic tail area by 18F-DOPA PET/CT. Distal (left-sided) pancreatectomy with resection of the body and tail led to cure. The resected specimen had the highest islet volume fraction and islet clustering being 4-fold compared to control. No mutations were detected in blood, whole-pancreatic tissue or in isolated islets. Despite negative BWS genetic investigations in the blood, somatic overgrowth DNA variants in the endocrine tissue of the left part of the pancreas may have been undetected.

### Histology

In the pancreatic specimens of our five patients, one or few pancreatic lobules had larger islets, while smaller islets were present throughout the resected pancreas, accompanied by clusters of endocrine cells. In 2011, Sempoux et al. described 16 histologically atypical CHI patients with morphological mosaicism of pancreatic islets, which lacked the histological features of classical diffuse or focal CHI (19). Two types of islets were reported: Large and small islets. Large, hyper-functional islets were confined to one or several adjacent lobules of the pancreas and around two-fold larger than small islets. Small islets were distributed throughout the entire pancreas (19). The histological picture in our five patients was somewhat reminiscent of the discrete histological changes described by Sempoux, even though the larger islets were not as clearly restricted two one or a few lobules. Besides, we detected a few scattered giant nuclei in endocrine cells in all cases.

### Non-coding *HK1* genotype-histotype correlation

In the later report of Henquin et al., 2013 (20), *HK1* was expressed in the hyper-functional islets in five of the 6 patients. In 2022, 17 CHI patients with non-coding *HK1* genetic variants in heterozygous state, or mosaic in leukocyte DNA, were described by Wakeling et al. (24).

Histo-morphometric, our heterozygous *HK1* Patient 1 had larger islets, but not a high islet density, compared to our somatic mosaic *HK1* Patients 2 and 3 (**Table 2**). Indeed, Patient 3 with “islet-only mosaic HK1-CHI” had the highest islet density, 4-fold compared to our control and 2 to 3-fold compared to Patient 1 and 2.

Rare giant cell nuclei were found in all five patients. Nuclear enlargement was seen in 0.8% of islets in one heterozygous HK1-CHI patient in the Wakeling et al. report (24), compared to 4.9% in an ABCC8-CHI control. The lower occurrence of giant nuclei may represent a relatively mild hyperinsulinism phenotype compared to K_ATP_-channel CHI.

Both our heterozygous and genetically mosaic HK1-CHI were associated with morphological mosaicism. This highlights that histological mosaicism only in some, but not all cases, correlate with genetic mosaicism. Moreover, the histo-morphometric variation in HK1-CHI could not be correlated to the genetic state of somatic mosaicism vs. heterozygosity.

### Histotype-phenotype correlations

Partial pancreatic resection did not lead to instant cure of the hyperinsulinism in our Patients 1-4, suggesting more widespread pathological changes in the pancreas as also suggested by the diffuse 18F-DOPA tracer uptake. While it is reasonable to hypothesize that our heterozygous HK1-CHI and CACNA1D patients had changes reminiscent of Sempoux’ morphological mosaicism of islets throughout the entire pancreas, it is not possible to determine whether the genetically mosaic HK1-CHI patients had widespread or localized changes without cure after 50-80% pancreatic resection. Hence, presumed localized histological changes, as also seen in LINE in patients with mosaic *ABCC8* or *GCK* mutations (21), may in fact represent scattered changes in a few lobules throughout the pancreas after low-grade mosaic mutations, especially in patients with lack of cure after resection of a smaller pancreatic segment.

### Non-coding *HK1* genotype details

Our genetic investigations showed that non-coding *HK1* variants may be absent in germline, but present in low-grade mosaic form in blood and pancreatic tissue (Patient 2), or even in islets only (Patient 3). Others have found low-grade somatic mutations in *ABCC8* or *GCK* in isolated islets of two CHI patients as well as in their whole-pancreatic tissue (21, 41). To our best knowledge, low-grade genetic “islet-only mosaicism” as seen in our Patient 3 has never been reported, neither for *HK1* or other genes related to CHI. In perspective, genetic investigations on isolated islets hold potential to identify genetic causes in more CHI patients with unknown genetics from germline or whole pancreatic tissue analyses.

Nucleotide changes have previously been reported in CHI patients (24, 33, 42) in the same non-coding *HK1* positions 10:g.69,348,891 and 10:g.69.348.896 as seen in our study (of note with use of genome reference GRCh37/hg19 in the cited papers and CRCh38/hg38 in our study). The 10:g.69,348,891C>T change is one of three changes previously seen in this position. The A>T change in position 10:g.69.348.896 in our islet-only mosaic HK1-CHI patient was novel and the third reported nucleotide change reported in this position. Our findings underscore the likely pathogenicity related to substitutions at these nucleotide positions.

When examining the GeTex, ENSEMBL and UCSC databases, we identified other motifs related to *HK1* promoters and enhancers than those reported by Wakeling et al. (24). Applying the filters for ENCODE cCREs, ENCODE Regulation, CpG, FANTOM5, GeneHancer, GTEX cis-eQTLs, JASPER Transcription Factors, ORegAnno, RefSeq FuncElemens, VISTA Enhancers in the UCSC browser, no regulatory elements were identified (**Figure 6A**). Analyzing the specified area for DNase Hypersensitive sites of the endocrine pancreas with H3K4me3 as a target, no such sites were observed in the ENCODEPROJECT (www.encodeproject.org). Trimethylation of histone H3 at lysine 4 (H3K4me3) is an epigenetic mark primarily associated with the promoter regions of actively transcribed genes. The presence of H3K4me3 at a gene’s promoter is a hallmark of active transcription. Our observed absence of a methylation site in the area points to a mechanism not involving promotor regulation at the transcriptional level as previously suggested.

When further analyzing RNAseq data in bTC3 cells (GSE249790; GSE248349) representing Mus Musculus and beta-cells from human and mice islets, a low but significant expression of the disallowed genes *HK1*, *LDHA*, and *SLC6A1* plus the canonical *PDX1*, *ISL1* and *NKX6.1* was observed. Using ESEfinder, release 3.0 (https://esefinder.ahc.umn.edu), the genetic variants in the non-coding *HK1* region in our study was found to cover exon splicing enhancers for SF2/ASF and SRp40 with both introduced variants causing diminished splicing activity (**Figure 6B**). Recently it has been shown that Exon-Mediated Activation of Transcription Starts (EMATS) govern the promotor usage by way of internal exon splicing. Hence, changing the splicing efficiency especially of highly included exons leads to alternative promotor usage.

Using data from GTEx (https://www.gtexportal.org), the *HK1* exon 2 can be seen to be part of the majority of known HK1 isoforms (**Figure 7A**). Thus, we hypothesize that the variants found in our patients cause usage of an alternative HK1 promotor leading to upregulating of activity bypassing the disallowed gene state, e.g. by promoting an alternative HK1 isoform. An alternative promotor at exon 2 is illustrated in **Figure 7B**). Applying EPDnew, a database of experimental validated promotors, in the UCSC genome browser, a candidate promoter can be visualized before exon 2 that in agreement with EMATs potentially could enhance the pancreatic islet specific isoform. Functional studies are warranted to clarify the impact of variants found in the non-coding *HK1* region.

### Strengths and limitations

This study had some limitations. FFPE tissue may contain artifacts that hamper in-depth sequencing (43). When performing laser-capture microdissection of islets of Langerhans, contribution of small amounts of exocrine cell DNA was unavoidable. Therefore, caution must be taken in the interpretation of the detected variants, especially regarding VAF. We found, however, *HK1* intron 2 variants in both a *de novo* heterozygous and low-grade mosaic form in leukocyte DNA, which supported our findings. We were not able to compare VAFs between areas with high and low beta cell volume fraction, as the tissue and islets for genetic analysis were selected at random throughout the specimen. Another limitation was our use of targeted NGS, where we only screened for a predetermined list of 140 genes.

Finally, our study on atypical CHI was only based on five patients and one control without precise age-match, given the rarity of such specimens. More detailed studies on atypical CHI genotype-histotype correlations, 18F-DOPA PET/CT findings and the effect of pancreatic surgery are warranted.

Strengths of our study included our morphometric histological analysis and the genetic analysis on isolated islets, enabling detection of a low-grade mosaic gene variant, undetectable in whole pancreatic tissue. Furthermore, we avoided PCR duplicates by adding UMI to sequencing libraries before undertaking any PCR amplification steps. This enabled the accurate bioinformatic identification of PCR duplicates that can be removed. By power calculation, our average sequencing depth of 1.733 allowed for detection of a VAF down to 0.5%. The validity of our patients’ VAF in the range of 0.9-1.1% was further strengthened by the complete lack of the DNA variants in our control tissue. In comparison, Boodhansingh et al. (21) had a detection threshold of 2% by NGS with a much higher average coverage of 16.835x, but seemingly without using UMI. They did, however, report a somatic *ABCC8* variant in blood and whole pancreatic tissue in 0.2-0.34% of base calls in a patient with LINE-CHI, significantly above the miscall read in controls.

## Conclusion

In conclusion, our surgical pancreas specimens from CHI patients with atypical histology had occurrence of larger islets in certain areas, while smaller islets were distributed throughout the pancreas. This was somewhat reminiscent of the so-called morphological mosaicism of pancreatic islets as per Sempoux et al. (19). The larger islets were, however, not entirely restricted to one or a few lobules. In addition, occurrence of enlarged nuclei were found in a few large islets in each case, but at a much lower frequency as in classical K_ATP_-channel diffuse CHI. Clinically, the patients had lower birth weight and later clinical presentation compared to classical K_ATP_-channel CHI patients. At the genetic level, heterozygous or low-grade mosaic *HK1* intron 2 DNA variants, or a *CACNA1D* frameshift mutation, were found. Genetic analysis in laser-capture microdissected islets of Langerhans allowed detection of a low-grade mosaic *HK1* variant, undetectable in blood and whole-pancreatic tissue. In perspective, genetic analysis focusing on islets may increase the detection rate of genetic causes to atypical CHI.

## Data Availability

The original contributions presented in the study are included in the article/**Supplementary Material**. Further inquiries can be directed to the corresponding author.

## References

[1] Yau D Laver TW Dastamani A Senniappan S Houghton JAL Shaikh G . Using referral rates for genetic testing to determine the incidence of a rare disease: The minimal incidence of congenital hyperinsulinism in the UK is 1 in 28,389. PLoS One. (2020) 15:e0228417. doi: 10.1371/journal.pone.0228417, PMID: 32027664 PMC7004321

[2] Hewat TI Johnson MB Flanagan SE . Congenital hyperinsulinism: current laboratory-based approaches to the genetic diagnosis of a heterogeneous disease. Front Endocrinol. (2022) 13:873254. doi: 10.3389/fendo.2022.873254, PMID: 35872984 PMC9302115

[3] Snider KE Becker S Boyajian L Shyng SL MacMullen C Hughes N . Genotype and phenotype correlations in 417 children with congenital hyperinsulinism. J Clin Endocrinol Metab. (2013) 98:E355–63. doi: 10.1210/jc.2012-2169, PMID: 23275527 PMC3565119

[4] Kapoor RR Flanagan SE Arya VB Shield JP Ellard S Hussain K . Clinical and molecular characterisation of 300 patients with congenital hyperinsulinism. Eur J Endocrinol. (2013) 168:557–64. doi: 10.1530/EJE-12-0673, PMID: 23345197 PMC3599069

[5] Yau D Stanley CA . Diazoxide-responsive forms of congenital hyperinsulinism. In: De Léon-Crutchlow DD Stanley CA , editors. Congenital hyperinsulinism A practical guide to diagnosis and management. Humana Press, Cham, Switzerland (2019). p. p.15–32.

[6] De Leon DD Arnoux JB Banerjee I Bergadá I Bhatti T Conwell LS . International guidelines for the diagnosis and management of hyperinsulinism. Horm Res Paediatr. (2024) 97:279–98. doi: 10.1159/000531766, PMID: 37454648 PMC11124746

[7] Zenker M Mohnike K Palm K . Syndromic forms of congenital hyperinsulinism. Front Endocrinol. (2023) 14:1013874. doi: 10.3389/fendo.2023.1013874, PMID: 37065762 PMC10098214

[8] de Lonlay-Debeney P Poggi-Travert F Fournet JC Sempoux C Dionisi Vici C Brunelle F . Clinical features of 52 neonates with hyperinsulinism. New Engl J Med. (1999) 340:1169–75. doi: 10.1056/NEJM199904153401505, PMID: 10202168

[9] Damaj L le Lorch M Verkarre V Werl C Hubert L Nihoul-Fékété C . Chromosome 11p15 paternal isodisomy in focal forms of neonatal hyperinsulinism. JClin Endocrinol Metab. (2008) 93:4941–7. doi: 10.1210/jc.2008-0673, PMID: 18796520

[10] Larsen AR Brusgaard K Christesen HT Detlefsen S . Genotype-histotype-phenotype correlations in hyperinsulinemic hypoglycemia. Histol Histopathol. (2024) 39:817–44. doi: 10.14670/HH-18-709, PMID: 38305063

[11] Gloyn AL Noordam K Willemsen MA Ellard S Lam WW Campbell IW . Insights into the biochemical and genetic basis of glucokinase activation from naturally occurring hypoglycemia mutations. Diabetes. (2003) 52:2433–40. doi: 10.2337/diabetes.52.9.2433, PMID: 12941786

[12] Cuesta-Muñoz AL Huopio H Otonkoski T Gomez-Zumaquero JM Näntö-Salonen K Rahier J . Severe persistent hyperinsulinemic hypoglycemia due to a *de novo* glucokinase mutation. Diabetes. (2004) 53:2164–8. doi: 10.2337/diabetes.53.8.2164, PMID: 15277402

[13] Wabitsch M Lahr G Van de Bunt M Marchant C Lindner M von Puttkamer J . Heterogeneity in disease severity in a family with a novel G68V GCK activating mutation causing persistent hyperinsulinaemic hypoglycaemia of infancy. Diabetes Med. (2007) 24:1393–9. doi: 10.1111/j.1464-5491.2007.02285.x, PMID: 17976205

[14] Kassem S Bhandari S Rodríguez-Bada P Motaghedi R Heyman M García-Gimeno MA . Large islets, beta-cell proliferation, and a glucokinase mutation. New Engl J Med. (2010) 362:1348–50. doi: 10.1056/NEJMc0909845, PMID: 20375417

[15] Stanley CA . Hyperinsulinism/hyperammonemia syndrome: insights into the regulatory role of glutamate dehydrogenase in ammonia metabolism. Mol Genet Metab. (2004) 81 Suppl 1:S45–51. doi: 10.1016/j.ymgme.2003.10.013, PMID: 15050973

[16] De Lonlay P Benelli C Fouque F Ganguly A Aral B Dionisi-Vici C . Hyperinsulinism and hyperammonemia syndrome: report of twelve unrelated patients. Pediatr Res. (2001) 50:353–7. doi: 10.1203/00006450-200109000-00010, PMID: 11518822

[17] Rahier J Guiot Y Sempoux C . Morphologic analysis of focal and diffuse forms of congenital hyperinsulinism. Semin Pediatr Surg. (2011) 20:3–12. doi: 10.1053/j.sempedsurg.2010.10.010, PMID: 21185997

[18] Kostopoulou E Dastamani A Güemes M Clement E Caiulo S Shanmugananda P . Syndromic forms of hyperinsulinaemic hypoglycaemia-A 15-year follow-up study. Clin Endocrinol. (2021) 94:399–412. doi: 10.1111/cen.14393, PMID: 33345357

[19] Sempoux C Capito C Bellanne-Chantelot C Verkarre V de Lonlay P Aigrain Y . Morphological mosaicism of the pancreatic islets: a novel anatomopathological form of persistent hyperinsulinemic hypoglycemia of infancy. J Clin Endocrinol Metab. (2011) 96:3785–93. doi: 10.1210/jc.2010-3032, PMID: 21956412

[20] Henquin JC Sempoux C Marchandise J Godecharles S Guiot Y Nenquin M . Congenital hyperinsulinism caused by hexokinase I expression or glucokinase-activating mutation in a subset of β-cells. Diabetes. (2013) 62:1689–96. doi: 10.2337/db12-1414, PMID: 23274908 PMC3636634

[21] Boodhansingh KE Yang Z Li C Chen P Lord K Becker SA . Localized islet nuclear enlargement hyperinsulinism (LINE-HI) due to ABCC8 and GCK mosaic mutations. Eur J Endocrinol. (2022) 187:301–13. doi: 10.1530/EJE-21-1095, PMID: 35674212 PMC9339501

[22] Boodhansingh KE Rosenfeld E Lord K Adzick NS Bhatti T Ganguly A . Mosaic GLUD1 mutations associated with hyperinsulinism hyperammonemia syndrome. Horm Res Paediatr. (2022) 95:492–8. doi: 10.1159/000526203, PMID: 35952631 PMC9671865

[23] Boodhansingh KE Lord K Adzick NS Bhatti T Ganguly A Stanley CA . Low-level mosaic GCK mutations in children with diazoxide-unresponsive congenital hyperinsulinism. J Clin Endocrinol Metab. (2024) 110:1923–8. doi: 10.1210/clinem/dgae713, PMID: 39382384 PMC12187123

[24] Wakeling MN Owens NDL Hopkinson JR Johnson MB Houghton JAL Dastamani A . Non-coding variants disrupting a tissue-specific regulatory element in HK1 cause congenital hyperinsulinism. Nat Genet. (2022) 54:1615–20. doi: 10.1038/s41588-022-01204-x, PMID: 36333503 PMC7614032

[25] Siersbæk J Larsen AR Nybo M Christesen HT . A sensitive plasma insulin immunoassay to establish the diagnosis of congenital hyperinsulinism. Front Endocrinol. (2020) 11:614993. doi: 10.3389/fendo.2020.614993, PMID: 33679602 PMC7935514

[26] Rasmussen AG Melikian M Globa E Detlefsen S Rasmussen L Petersen H . The difficult management of persistent, non-focal congenital hyperinsulinism: A retrospective review from a single, tertiary center. Pediatr Diabetes. (2020) 21:441–55. doi: 10.1111/pedi.12989, PMID: 31997554

[27] Christiansen CD Petersen H Nielsen AL Detlefsen S Brusgaard K Rasmussen L . 18F-DOPA PET/CT and 68Ga-DOTANOC PET/CT scans as diagnostic tools in focal congenital hyperinsulinism: a blinded evaluation. Eur J Nucl Med Mol Imaging. (2018) 45:250–61. doi: 10.1007/s00259-017-3867-1, PMID: 29116340 PMC5745571

[28] Globa E Christesen HT Mortensen MB Houghton JAL Nielsen AL Detlefsen S . Congenital hyperinsulinism in the Ukraine: a 10-year national study. Front Endocrinol. (2024) 15:1497579. doi: 10.3389/fendo.2024.1497579, PMID: 39741883 PMC11686448

[29] Bankhead P Loughrey MB Fernández JA Dombrowski Y McArt DG Dunne PD . QuPath: Open source software for digital pathology image analysis. Sci Rep. (2017) 7:16878. doi: 10.1038/s41598-017-17204-5, PMID: 29203879 PMC5715110

[30] Christesen HT Christensen LG Löfgren ÅM Brøndum-Nielsen K Svensson J Brusgaard K . Tissue variations of mosaic genome-wide paternal uniparental disomy and phenotype of multi-syndromal congenital hyperinsulinism. Eur J Med Genet. (2020) 63:103632. doi: 10.1016/j.ejmg.2019.02.004, PMID: 30797057

[31] Wieland I Schanze I Felgendreher IM Barthlen W Vogelgesang S Mohnike K . Integration of genomic analysis and transcript expression of *ABCC8* and *KCNJ11* in focal form of congenital hyperinsulinism. Front Endocrinol. (2022) 13:1015244. doi: 10.3389/fendo.2022.1015244, PMID: 36339418 PMC9634566

[32] Bendix J Laursen MG Mortensen MB Melikian M Globa E Detlefsen S . Intraoperative ultrasound: A tool to support tissue-sparing curative pancreatic resection in focal congenital hyperinsulinism. Front Endocrinol. (2018) 9:478. doi: 10.3389/fendo.2018.00478, PMID: 30186238 PMC6113400

[33] Bennett JJ Saint−Martin C Neumann B Mannisto JME Houghton JAL Empting S . Non−coding *cis*−regulatory variants in HK1 cause congenital hyperinsulinism with variable disease severity. Gen Med. (2025) 17:17. doi: 10.1186/s13073-025-01440-w, PMID: 40033430 PMC11874398

[34] Becker TC BeltrandelRio H Noel RJ Johnson JH Newgard CB . Overexpression of hexokinase I in isolated islets of Langerhans via recombinant adenovirus. Enhancement of glucose metabolism and insulin secretion at basal but not stimulatory glucose levels. J Biol Chem. (1994) 269:21234–8. doi: 10.1016/S0021-9258(17)31953-1, PMID: 8063745

[35] Flanagan SE Vairo F Johnson MB Caswell R Laver TW Lango Allen H . A CACNA1D mutation in a patient with persistent hyperinsulinaemic hypoglycaemia, heart defects, and severe hypotonia. Pediatr Diabetes. (2017) 18:320–3. doi: 10.1111/pedi.12512, PMID: 28318089 PMC5434855

[36] De Mingo Alemany MC Mifsud Grau L Moreno Macián F Ferrer Lorente B León Cariñena SA . *de novo* CACNA1D missense mutation in a patient with congenital hyperinsulinism, primary hyperaldosteronism and hypotonia. Channels. (2020) 14:175–80. doi: 10.1080/19336950.2020.1761171, PMID: 32336187 PMC7219433

[37] Kummer S Rinné S Seemann G Bachmann N Timothy K Thornton PS . Hyperinsulinemic hypoglycemia associated with a ca(V)1.2 variant with mixed gain- and loss-of-function effects. Int JMolSci. (2022) 23:8097. doi: 10.3390/ijms23158097, PMID: 35897673 PMC9332183

[38] Kessi M Chen B Peng J Yan F Yang L Yin F . Calcium channelopathies and intellectual disability: a systematic review. Orphanet J Rare Dis. (2021) 16:219. doi: 10.1186/s13023-021-01850-0, PMID: 33985586 PMC8120735

[39] Wang H Zhua J-K Chenga L Maoa G Chena H Wua X . Dominant role of CACNA1D exon mutations for blood pressure regulation. J Hypertens. (2022) 40:819–34. doi: 10.1097/HJH.0000000000003085, PMID: 35142739

[40] Stanton AM Heydarpour M Williams JS Williams GH Adler GK . *CACNA1D* gene polymorphisms associate with increased blood pressure and salt sensitivity of blood pressure in white individuals. Hypertension. (2023) 80:2665–73. doi: 10.1161/HYPERTENSIONAHA.123.21229, PMID: 37846579 PMC10843263

[41] Li C Juliana CA Yuan Y Li M Lu M Chen P . Phenotypic characterization of congenital hyperinsulinism due to novel activating glucokinase mutations. Diabetes. (2023) 72:1809–19. doi: 10.2337/db23-0465, PMID: 37725835 PMC10658072

[42] Velde CD Molnes J Berland S Njølstad PR Molven A . Clinical and genetic characteristics of congenital hyperinsulinism in Norway: A nationwide cohort study. J Clin Endocrinol Metab. (2025) 110:554–63. doi: 10.1210/clinem/dgae459, PMID: 38963811 PMC11747666

[43] Do H Dobrovic A . Sequence artifacts in DNA from formalin-fixed tissues: causes and strategies for minimization. Clin Chem. (2015) 61:64–71. doi: 10.1373/clinchem.2014.223040, PMID: 25421801

