# Supplementary material for: Germline and somatic mutations in histologically atypical congenital hyperinsulinism
Source: Front Endocrinol (Lausanne). 2026 Jan 5;16:1692539. doi: 10.3389/fendo.2025.1692539 (PMC12812534; doi:10.3389/fendo.2025.1692539)
Supplement: Supplementary file 1 [file DataSheet1.docx]

**Supplementary Information**

**Germline and somatic mutations in histologically atypical congenital hyperinsulinism**

**AUTHORS**

Annette Rønholt Larsen^a,b,c,d^ Evgenia Globa^e^, Ditte Caroline Andersen^b,f^, Catarina Limbert^g^, Åsa Mattsson Löfgren^h^, Anne Lerberg Nielsen^i^, Michael Bau Mortensen^c,j^, Eva Kildall Hejbøl^k^, Klaus Brusgaard^b,c,d,l,m†^, Sönke Detlefsen^b,c,k†^, Henrik Thybo Christesen^a,b,c,l†^

^a^ Hans Christian Andersen Children’s Hospital, Odense University Hospital, Odense, Denmark

^b^ Department of Clinical Research, Faculty of Health Sciences, University of Southern Denmark, Denmark

^c^ Odense Pancreas Center (OPAC), Odense University Hospital, Odense, Denmark

^d^ Department of Clinical Genetics, Odense University Hospital Odense, Denmark

^e^ Department of Pediatric Endocrinology, Ukrainian Scientific and Practical Center of Endocrine Surgery, Transplantation of Endocrine Organs and Tissues of the Ministry of Health of Ukraine, Kyiv, Ukraine

^f^ DCA-group, University of Southern Denmark; Dep. Clinical Biochemistry, Odense University Hospital, Denmark.

^g^ Unit for Pediatric Endocrinology and Diabetes, CHLC, Hospital Dona Estefania, Lisbon, Portugal

^h^ Department of Pediatrics, Helsingborg Hospital, Sweden

^i^ Department of Nuclear Medicine, Odense University Hospital, Odense, Denmark

^j^ Upper Gastrointestinal and Hepato-Pancreato-Biliary Section, Department of Surgery, Odense University Hospital, Odense, Denmark

^k^ Department of Pathology, Odense University Hospital, Odense, Denmark

^l^ Steno Diabetes Center, Odense University Hospital, Odense, Denmark

^m^ Department of Regional Health Research, University of Southern Denmark, Denmark

^†^ These authors share last authorship

**Supplementary Table 1.** Overview of antibodies and conditions for immunostaining

| **Antibody,**  **clone** | **Platform** | **Incubation/**  **demasking** | **Dilution** | **Company + code** |
| --- | --- | --- | --- | --- |
| p57KIP2,  KP39 | BenchMark | 24 minutes /  32 minutes | 1:800 | LabVision  MS-897-P0 |
| Insulin,  2D11-H5 | BenchMark | 24 minutes /  16 minutes | 1:2000 | Santa Cruz  Sc-8033 |
| Glucagon,  EP74 | BenchMark | 8 minutes /  32 minutes | 1:200 | Cell Marque  AC-0074A |
| Somatostatin, EP130 | BenchMark | 32 minutes /  32 minutes | 1:250 | Cell Marque  332R-14 |
| Synaptophysin, 27G12 | BenchMark | 32 minutes /  48 minutes | 1:50 | NovoCastra  27G12 |

All antibodies were incubated at 36 °C. Demasking was performed in CC1: Cell Conditioning solution 1 (pH 8.5) at 100 °C. BenchMark Ultra, Ventana, Roche was used with the OptiView-DAB detection kit, Ventana Medical Systems, Tucson, AZ.

**Supplementary Table 2.** Targeted gene panel

| *ABCC8, ACAT1, ACSF3, ADCY7, AGL, AGPAT2, AIRE, AKT2, ALDOA, ALDOB, APPL1, BSCL2, BTD, BAAT, CACNA1C, CACNA1D, CEL, CISD2, COQ2, COQ9, CPT1A, CTLA4, DCAF17, DNAJC3, DYRK1B, EDEM2, EIF2AK3, EIF2S3, ENO3, EPM2A, FBP1, FBP2, FOXA2, FOXP3, G6PC, GATA4, GATA6, GBE1, GCK, GLIS3, GLUD1, GPC3, GYG1, GYG2, GYS1, GYS2, GAA, HADH, HK1, HLCS, HMGCL, HMGCS2, HNF1A, HNF1B, HNF4A, IER3IP1, IGF2BP1, IL2RA, INS, INSR, IPEX, ITCH, IVD, JAK1, KCNJ11, KCNQ1, KDM6A, KMT2D, LAMP2, LDHA, LDHB, LMNA, LPL, LRBA, MAFA, MAGEL2, MCEE, MNX1, MPV17, MUT, NCOR1, NEK11, NEUROD1, NEUROG3, NHLRC1, NKX2-2, NSD1, OXCT1, PAX6, PC, PCBD1, PCCA, PCCB, PCK1, PCK2, PDX1, PFKL, PFKM, PGAM2, PGK1, PGM1, PHKA1, PHKA2, PHKB, PHKG1, PHKG2, PIK3R1, PLIN1, PMM2, POLD1, PPARG, PPP1R15B, PRKAG2, PRKAG3, PTF1A, PYGL, PYGM, RBCK1, RFX6, RNF40, RYR3, SIRT1, SLC16A1, SLC19A2, SLC25A20, SLC27A5, SLC29A3, SLC2A2, SLC37A4, SLC5A2, STAT1, STAT3, STAT5B, TANGO2, TNFAIP3, TRMT10A, UCP2, WFS1, ZBTB20, ZFP57,* GRCh38/hg38 chr10:69,348,884-69,348,931 HK1 non-coding region |
| --- |

**Supplementary Figure 1**. Sanger Sequencing shows a de novo heterozygous mutation in Patient 1, C >T, position 10:g.69,348,891 in HK1 non-coding region.


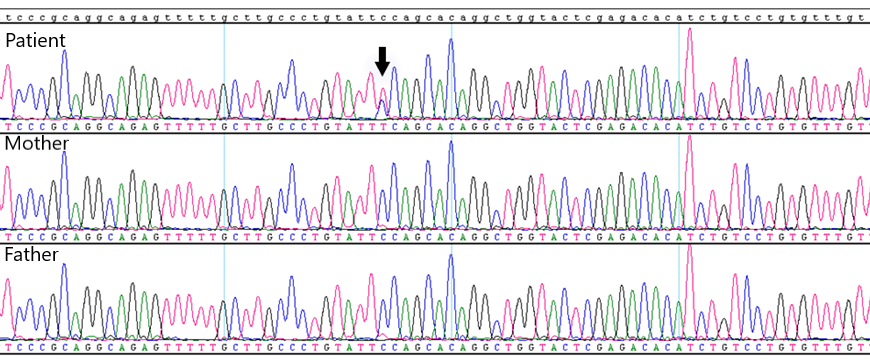


The PCR reaction was performed with KAPA2G Robust HotStart ReadyMIX (KAPA Biosystems, Inc.) and with primers from Pxlence (Pxlence, Inc. The Primers were designed for the non-coding HK1 region*.* The Primers are: 1) forward: HK1-Forward: AGCCTGGGCAACAGAAAC and HK1- and 2) reverse: GCTACAAGCTCAGCCTCTTTC. Sanger Sequencing was performed on ABI 3730xl DNA Analyser (Applied Biosystems, Inc.). The program DNASTAR Sequencing Analyser (DNASTAR, Inc.) was used following the protocol to analyse the Sanger Sequencing data.
